# Supplementary material for: The Male Mouse Meiotic Cilium Emanates from the Mother Centriole at Zygotene Prior to Centrosome Duplication
Source: Cells. 2022 Dec 29;12(1):142. doi: 10.3390/cells12010142 (PMC9818220; doi:10.3390/cells12010142)
Supplement: Supplementary file 1 [file cells-12-00142-s001.zip › cells-2125991-supplementary.pdf]

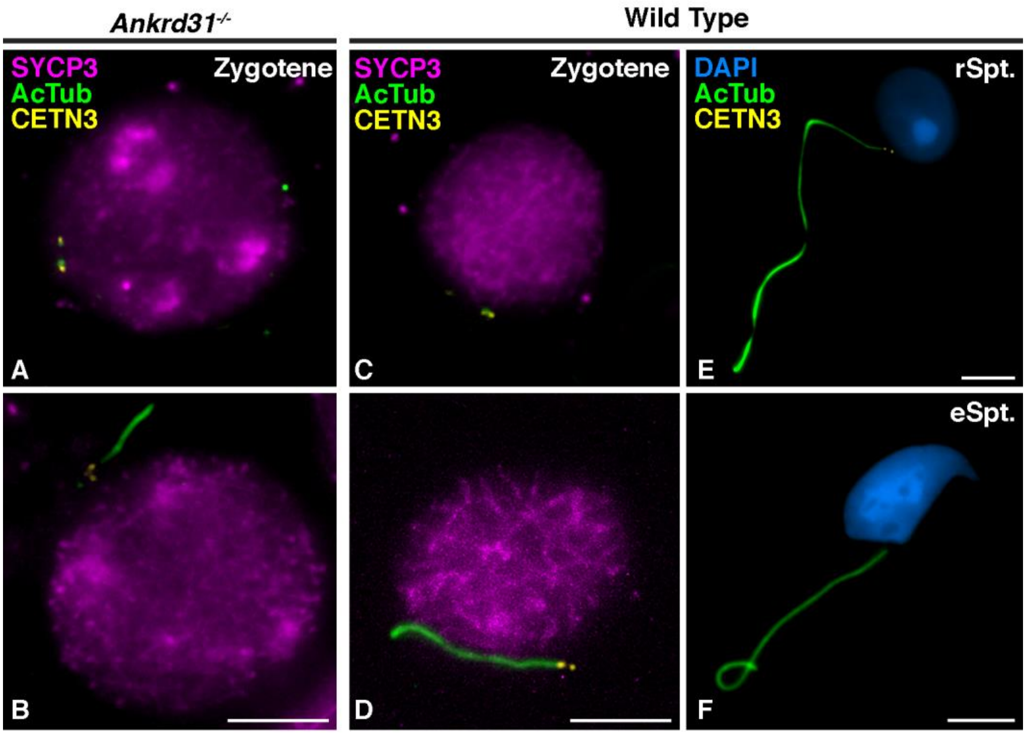

Supplementary Figure S1. Detection of the cilia in *Ankrd31*$^{-/-}$ sterile mice and WT spermatocytes. Triple immunolabelling of SYCP3 (magenta), acetylated Tubulin (AcTub) (green) and Centrin 3 (CETN3) (yellow) on mouse spermatocytes of *Ankrd3*$^{-/-}$ (A,B) at (A) zygotene, and (B) zygotene presenting cilium. Mouse spermatocytes of WT (C,D) at (C) zygotene, and (D) zygotene presenting cilium, (E)€ Early round spermatid (rSpt.) and (F) mature elongated spermatid (eSpt.). Scale bar in B, D, E and F represent 5 µm.

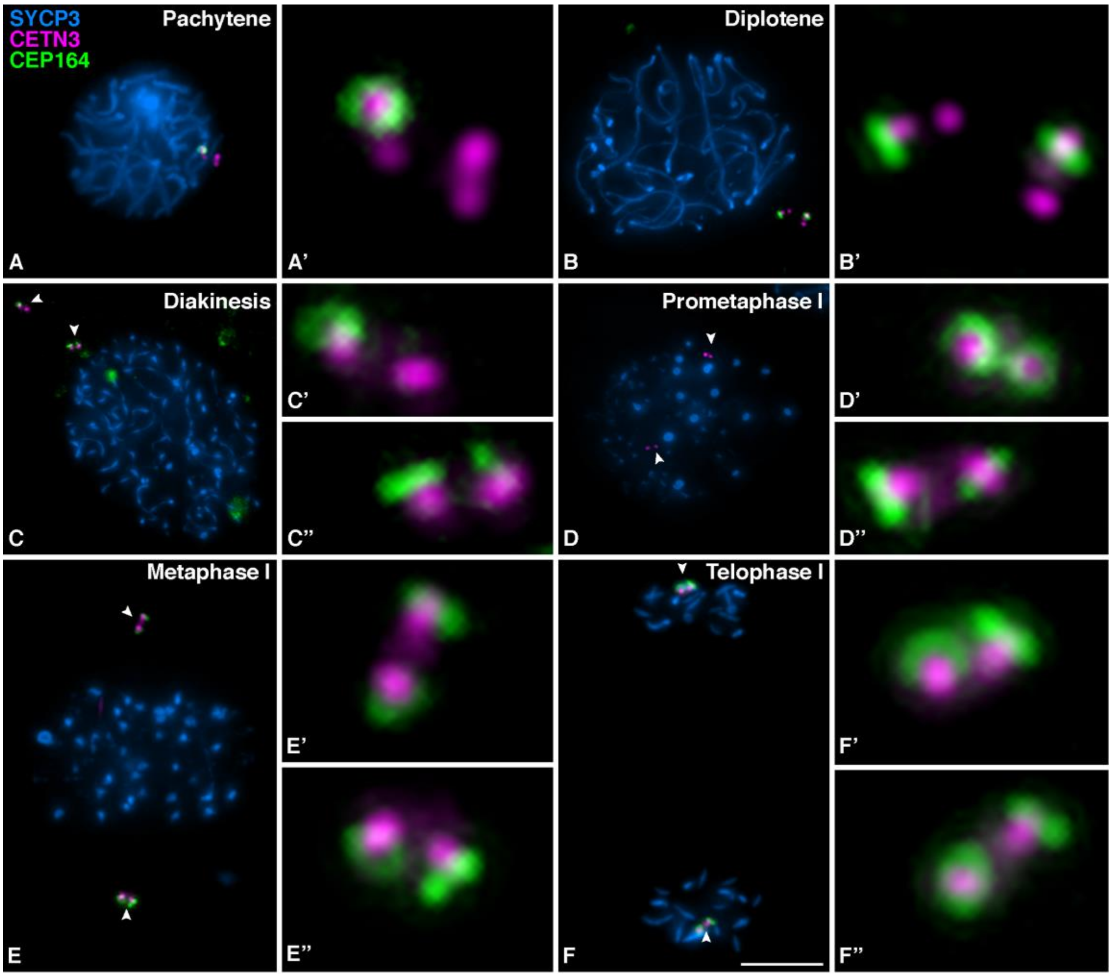

Supplementary Figure S2. Distribution of CEP164 in mouse spermatocytes during the first meiotic division. Triple immunolabelling of SYCP3 (blue), Centrin 3 (CETN3) (magenta) and CEP164 (green) in mouse spermatocytes at (A) Pachytene (B) Diplotene, (C) Diakinesis, (D) Prometaphase I, (E) Metaphase I, (F) Telophase I. For images A´-F´ the 300X magnification of the centrosomes is shown (C′-C′′, D′-D′′, E′-E′′ and F′-F′′, white arrowheads). Scale bar in F represents 5 μm.

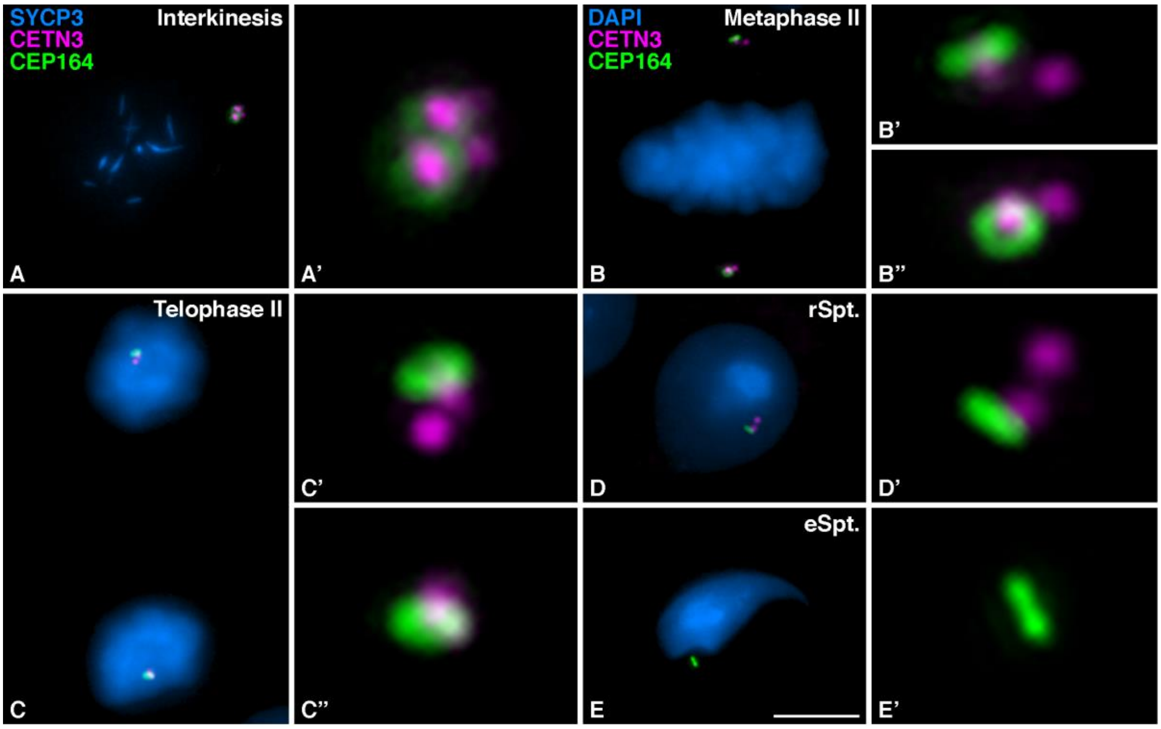

Supplementary Figure S3. Distribution of CEP164 in mouse spermatocytes during the second meiotic division and spermiogenesis. Triple immunolabelling of SYCP3 (blue), Centrin 3 (CETN3) (magenta) and CEP164 (green) on squashed WT mouse spermatocytes at (A) Interkinesis. And double immunolabelling of Centrin 3 (CETN3) (magenta) and CEP164 (green), with chromatin stained with DAPI (blue) at (B) Metaphase II, (C) Telophase II (D) Early round spermatid (rSpt.) and mature elongated spermatid (eSpt.). For images A´-E´ the 300X magnification of the centrosomes is shown. Scale bar in E´ represents 5 μm.
